# Kelvin probe force microscopy maps the chiral-induced spin selectivity effect in chiral halide perovskites

**DOI:** 10.1093/nsr/nwaf456

**Published:** 2025-10-24

**Authors:** Sebastian Fernández, Manchen Hu, Daniel N Congreve

**Affiliations:** Department of Electrical Engineering, Stanford University, USA; Department of Electrical Engineering, Stanford University, USA; Department of Electrical Engineering, Stanford University, USA

Recently, the incorporation of chiral ligands within halide perovskites has yielded both spin-polarized currents and chiral light emission—unlocking new possibilities for opto-spintronic devices based on this semiconductor class [1]. However, the spatial variability at the nanoscale, as well as its consequences on spin transport, in these chiral halide perovskite (CHP) materials remains an open question. In a recent study published in *National Science Review*, Li and co-workers employed Kelvin probe force microscopy (KPFM) to map the chiral-induced spin selectivity (CISS) effect with nanoscale resolution within CHP thin films [2]. This method offers a nanoscale viewpoint into the CISS effect within CHPs, which could lead to new observations regarding the mechanisms that drive the CISS effect.

**Figure 1.**
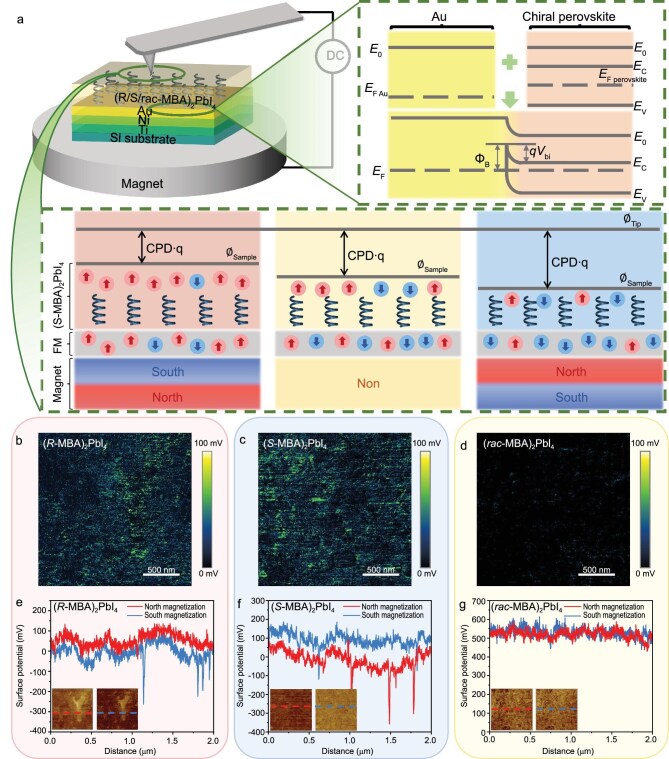
KPFM maps the CISS effect in CHPs at the nanoscale. (a) KPFM experimental setup schematic and CISS-detecting principle. 2D maps (b–d) and line profiles (e–g) of the contact potential differences between the north- and south-pole magnetizations of (*R-/S-/rac-*MBA)_2_PbI_4_ thin films. Dashed lines in the insets of (e–g) indicate where the line profiles were measured. Adapted from Li and co-workers [2].

Previous characterizations of the CISS effect on CHPs have largely relied on magnetic conductive probe atomic force microscopy (mCP-AFM), which employs a small magnetized probe that measures current–voltage (I–V) characteristics from the CHP. By magnetizing the probe and changing the chirality of the CHP, different I–V curves emerge, which can be attributed to spin-polarized charge carriers, and spin polarizations greater than 80% are often observed [1]. However, mCP-AFM measurements are challenging due to material degradation and relatively limited measurement areas. Alternatively, KPFM operates in tapping mode and does not require magnetization of the probe, which can reduce measurement-induced damage to the CHP thin film and simplify the mapping process. Li and co-workers spin-coated CHP thin films on a ferromagnetic substrate and placed them on top of a strong magnet, where a spin-Schottky junction was formed at the Au/CHP interface. The contact potential difference (CPD) was then deterministically altered depending on both the polarization of the ferromagnetic substrate and chirality of the CHP. For example, Fig. 1a shows how the CPD changes for (*S-*MBA)_2_PbI_4_ across magnet polarity. To illustrate KPFM mapping, Li and co-workers showcased CPD difference images for (*R-/S-/rac-*MBA)_2_PbI_4_ thin films by subtracting north and south magnetization CPD mappings for each CHP thin film, pixel by pixel (Fig. 1b–d). Figure 1b and c show the nanometer-scale non-uniformity in spin charge transport for (*R-*MBA)_2_PbI_4_ and (*S-*MBA)_2_PbI_4_, respectively, while Fig. 1d shows that (*rac-*MBA)_2_PbI_4_ does not exhibit the CISS effect. Lastly, Fig. 1e–g shows the selected potential line profiles of (*R-/S-/rac-*MBA)_2_PbI_4_ thin films under different magnetization conditions, which further support the existence of non-uniform spin transport. These findings offer a perspective on the difference between reports of high spin polarization in CHPs at the nanoscale level (>80%) [3] and more modest polarizations at the macroscopic level.

The utility of KPFM for visualizing nanoscale CISS distributions in CHPs has been clearly demonstrated, although there are additional considerations for future work. For example, approaches that enhance the uniformity and morphological control of CHPs should be explored, as the observed nanoscale variations suggest that local inhomogeneities could play a decisive role in spin transport behavior. Such efforts would facilitate the collective understanding of structure–property–spin correlations in CHPs, opening the opportunity to accelerate CHP development for important applications in quantum computing, neuromorphic computing, virtual reality and more.
